# Editorial: Thrombo-inflammation in cardiovascular disease and health

**DOI:** 10.3389/fcvm.2023.1294874

**Published:** 2023-10-11

**Authors:** Richard G. Jung, Nicholas Kipshidze, Anne-Claire Duchez

**Affiliations:** ^1^ CAPITAL research group, Division of Cardiology, University of Ottawa Heart Institute, Ottawa, ON, Canada; ^2^ Division of Internal Medicine, The Ottawa Hospital, Ottawa, ON, Canada; ^3^ Division of Interventional Therapy, New York Cardiovascular Research, New York, NY, United States; ^4^ Hemostasis and Thrombosis Research Laboratories, Loyola University Medical Center, Chicago, IL, United States; ^5^ INSERM, U 1059 SAINBIOSE, Université Jean Monnet, Mines Saint-Étienne, Saint-Etienne, France; ^6^ Etablissement Français du Sang Auvergne-Rhône-Alpes, Research Department, Saint-Etienne, France

**Keywords:** thrombo-inflammation, cardiovascular disease, endothelium, platelet, DVT, COVID-19, vascular stent

**Editorial on the Research Topic** Thrombo-inflammation in cardiovascular disease and health

Thrombosis associated with inflammation (thrombo-inflammation) often occurs in a broad range of vascular diseases [such as venous thromboembolism (VTE), thrombophlebitis, atherosclerosis, and stroke], inflammatory diseases (such as diabetes and autoimmune conditions), malignancies, and infectious diseases including sepsis and COVID-19. This Research Topic on *Thrombo-inflammation in Cardiovascular Disease and Health* highlights some of the latest findings and evidence from investigations on thrombo-inflammation in atherosclerosis, VTE, COVID-19, thrombo-occlusive disease, and in cardiovascular health.

The collection begins with a comprehensive analysis conducted by Tan et al., who conducted a bibliometric assessment of scholarly publications pertaining to inflammasomes in the context of atherosclerosis published from 2002 to 2022. Inflammasome is a concept that was highlighted for the first time by Jürg Tshopp et al. (1) in 2002 and characterized as an intracellular complex of molecules including Caspase-1 and NALP1. Signals of stress activate inflammasomes that modulate inflammatory precursors participating in innate immune response and pathologies. This bibliometric evaluation was collected first with the Web of Science Core Collection, and 894 studies were identified and then analyzed and visualized with Citespace V and VOSviewer. Since 2008, there has been an increasing number of publications on inflammasome and atherosclerosis, with 62 countries and 338 institutions participating in the research field. These articles are published in 626 academic journals, with the top ten including Arteriosclerosis, Thrombosis, and Vascular Biology (ATVB), Atherosclerosis, and Circulation. The top three cited publications are published in Nature, Circulation, and ATVB.

The collection continues with a brief research report by Baksamawi et al*.* on the accumulation of platelet in an endothelium-coated elastic vein valve model of deep vein thrombosis (DVT). Venous thromboembolism (VTE) encompasses DVT, which is the formation of a thrombus in deep vein with low shear stress. Thrombus develops behind the venous valve leaflet, where blood may remain with accumulation of immune cells and platelets, a process preceding thrombus formation with clinical risk factors well characterized by Virchow's triad. Animal model use in research is complex from a methodological perspective, and reproducibility and ethics issues must also be taken into account (2). Based on this observation, the authors developed an “*in-vitro* novel microfluidics vein-on-chip with moving valve leaflets to mimic the hydrodynamics in a vein”. The chip was designed and produced with complex engineering. A monolayer of endothelial cells was coated on it. Briefly, they developed a microfluidic model with a flow pattern similar to that of venous conditions. Stimulation of platelets with thrombin led to platelet aggregation in the basal and luminal surface leaflet, depending on GP1bα-vwF interaction. Moreover, endothelial stimulation by histamine led to platelet aggregation behind the valve. In future studies, the impact on other key molecules such as tissue factor should be evaluated in this model.

Endothelium activation is led by different triggers, such as histamine, but also infectious etiologies. Ebermeyer et al. have presented, in a brief scientific report, the endothelium activation mechanism during coronavirus disease (COVID)-19 infection characterized by vascular inflammation. In this study, COVID-19 patient plasma was replaced by fresh non-contaminated plasma in order to remove inflammatory molecules such as immune complex and cytokines. The authors tested different plasmas from COVID-19 patients and from therapeutic plasma exchange (TPE) and then evaluated endothelial permeability and cytokine release. This study showed the benefit of removing inflammatory factors from the blood; however, TPE induced cellular activation, including platelet and endothelial activation, which affected its efficacy. During the pandemic, extensive research was conducted on different aspects of COVID-19. For example, neutrophil extracellular traps (NET) contribute to immune thrombosis in COVID-19. The review of Zdanyte et al. in our collection focused on the translational and clinical relevance of NET research in cardiovascular thrombo-occlusive diseases. They highlighted the involvement of NETosis in the pathophysiology of atherosclerosis, in some complications such as myocardial infarction and ischemic stroke, and in VTE.

The last publication from our collection was led by Simard et al. as an original research article on the involvement of a molecule (dipyridamole) in vascular healing following a stent implantation in a rabbit model. Percutaneous coronary intervention with stent implantation is still challenging due to stent-related adverse events, with the hyperproliferation of smooth muscle cells (SMC) resulting in in-stent restenosis. The authors evaluated the role of dipyridamole (DP) in an *in vivo* New Zealand white rabbit model with stents deployed in the abdominal aorta. They demonstrated a reduction in neointimal formation and increase in stent healing. Furthermore, they observed that DP reduces SMC proliferation and improves stent healing with anti-platelet therapies. This finding will need to be confirmed in a human study, but it is promising for stent healing, cardiovascular health, and quality of life for cardiovascular disease patients.

The guest editors express their anticipation that this study topic will offer readers novel insights into and views about thrombo-inflammation in cardiovascular disease and health, encompassing both fundamental and translational mechanisms. Taken together, our collection paves the way for new research using the *in vitro* microfluidics vein-on-chip with moving valve leaflets model developed by Baksamawi et al. to study molecules such as tissue factor, pro-inflammatory cytokines, and lipid mediators in different pathophysiologic contexts. Moreover, the therapeutic plasma transfer described in a COVID study by Ebermeyer et al. could be challenged with the study of other pro/anti-inflammatory molecules, the implication of this therapy on platelet activation, and the interaction of platelets with endothelium and other cell types. Last but not least, the study led by Simard et al. on dipyridamole will open a new way to study vascular healing after surgery or trauma.
